# Recent advances in injectable hydrogels for osteoarthritis treatments

**DOI:** 10.3389/fbioe.2025.1644222

**Published:** 2025-08-06

**Authors:** Jiayi Chen, Mingcong Deng, Jiangliang Wang, Yuanwen Liu, Ziran Hu, Feifan Luan, Huifeng Zhu, Chenxiao Zheng

**Affiliations:** ^1^ Zhongshan Hospital of Traditional Chinese Medicine Affiliated to Guangzhou University of Traditional Chinese Medicine, Zhongshan Hospital of Traditional Chinese Medicine, Zhongshan, Guangdong, China; ^2^ Shunde Hospital of Guangzhou University of Chinese Medicine, Foshan, Guangdong, China; ^3^ Department of Orthopedics, Liuyang Hospital of Traditional Chinese Medicine, Liuyang, Hunan, China; ^4^ Department of Orthopedics, Luoding Hospital of Traditional Chinese Medicine, Yunfu, Guangdong, China; ^5^ Beijing University of Chinese Medicine Shenzhen Hospital (Longgang), Shenzhen, Guangdong, China; ^6^ The Fifth Affiliated Hospital of Southern Medical University, Guangzhou, Guangdong, China

**Keywords:** injectable hydrogels, osteoarthritis, therapeutic hydrogels, biocompatibility, stimuli-responsive hydrogels

## Abstract

Osteoarthritis (OA) is a degenerative joint disease characterized by cartilage degradation, synovial inflammation, and subchondral bone alterations, poses significant challenges due to its high prevalence and associated disability. Injectable hydrogels have emerged as promising candidates for OA treatment due to their ability to deliver bioactive molecules directly to the affected joint, enhancing local efficacy while minimizing systemic side effects. This review focuses on recent advances in injectable hydrogels for OA treatment, emphasizing their structural design, functional properties, and therapeutic applications. We further discuss the advantages and limitations of natural, synthetic, and composite hydrogels, as well as innovative cross-linking strategies and stimuli-responsive behaviors. Thermosensitive, pH-responsive, enzyme-responsive, and multi-responsive hydrogels are highlighted for their potential to achieve intelligent drug delivery, inhibit cartilage degradation, and reduce inflammation. Overall, injectable hydrogels hold great promise for OA treatment and become an effective therapeutic option with further research and innovation.

## 1 Introduction

Osteoarthritis (OA) as a degenerative joint disease, poses significant challenges to global public health due to its high prevalence and associated disability (Chen X. et al., 2025; Darlow et al., 2025; Easwaran et al., 2024). It is characterized by the progressive degradation of articular cartilage, subchondral bone alterations, and synovial inflammation, leading to pain, stiffness, and reduced mobility (Kroon et al., 2016; Liew et al., 2023). The pathophysiology of OA involves multiple intertwined processes, including cartilage degradation, inflammatory responses, and abnormal changes in subchondral bone (Majeed et al., 2018; Sampath et al., 2023). Despite advances in understanding these mechanisms, current treatments primarily offer symptomatic relief rather than addressing the underlying causes. Non-steroidal anti-inflammatory drugs (NSAIDs), glucocorticoid injections, and physical therapy provide temporary pain relief but fail to halt or reverse the progression of cartilage degradation and inflammation (Tekeoğlu et al., 2024; Theeuwes et al., 2021). Moreover, long-term use of NSAIDs and glucocorticoids can lead to serious side effects, highlighting the urgent need for novel therapeutic strategies (Yao et al., 2024; Zhu et al., 2024).

Injectable hydrogels have emerged as promising candidates for OA treatment due to their ability to deliver bioactive molecules directly to the affected joint, thereby enhancing local efficacy while minimizing systemic side effects (Sarvepalli et al., 2025; Zhu S. et al., 2022). These hydrogels can be designed from natural polymers, synthetic polymers, or a combination thereof, each offering unique advantages. Natural polymer-based hydrogels, such as those derived from hyaluronic acid (HA), chitosan (CS), alginate, collagen, gelatin, and fibrinogen, exhibit excellent biocompatibility, biodegradability, and resemblance to the extracellular matrix (ECM) (Mellati et al., 2021; Salama et al., 2025; Sarvepalli and Kandagatla, 2025). HA, for example, plays a crucial role in maintaining joint lubrication and reducing friction (Kondiah et al., 2016; Kováč et al., 2023), whereas CS provides antibacterial properties and supports mesenchymal stem cell growth (Kondiah et al., 2016). Synthetic polymers like poly(N-isopropylacrylamide) (PNIPAm), polyethylene glycol (PEG), and poloxamers offer precise control over mechanical properties and drug release patterns through temperature-sensitive and stimuli-responsive behaviors (Jeznach et al., 2018). By combining the benefits of both natural and synthetic polymers, composite hydrogels aim to achieve enhanced mechanical strength, controlled degradation rates, and improved therapeutic outcomes (Gao et al., 2021; Ghandforoushan et al., 2023).

The design of injectable hydrogels for OA treatment encompasses various cross-linking strategies, including chemical, physical, and multiple cross-linking methods (von Lospichl et al., 2017; Xue et al., 2021). Chemical cross-linking, which involves the formation of covalent bonds using reagents like glutaraldehyde or EDC/NHS systems, offers high stability and controllable degradation rates (Song et al., 2022). However, it may introduce cytotoxicity issues. Physical cross-linking, relying on non-covalent interactions such as hydrogen bonding or ionic interactions, provides gentler conditions but often results in weaker mechanical properties (Singh et al., 2018). Multiple cross-linking approaches combine the strengths of both methods, enabling more flexible regulation of degradation and drug release behaviors. Stimuli-responsive hydrogels represent a significant innovation in OA therapy, as they can adapt to pathological changes in the joint (Kalairaj et al., 2024; Roffi et al., 2018). Thermosensitive injectable hydrogels, such as those based on poloxamer 407, undergo sol-gel transitions at body temperature, facilitating localized drug delivery (Gonzalez-Fernandez et al., 2022). pH-responsive hydrogels exploit the acidic OA microenvironment to trigger drug release, while enzyme-responsive hydrogels target overexpressed matrix metalloproteinases (MMPs) to achieve site-specific therapy (Chuah et al., 2017). Multi-responsive hydrogels, integrating temperature, pH, and enzyme sensitivity, further enhance therapeutic precision by addressing the multifaceted nature of OA (Chen et al., 2024).

**FIGURE 1:**
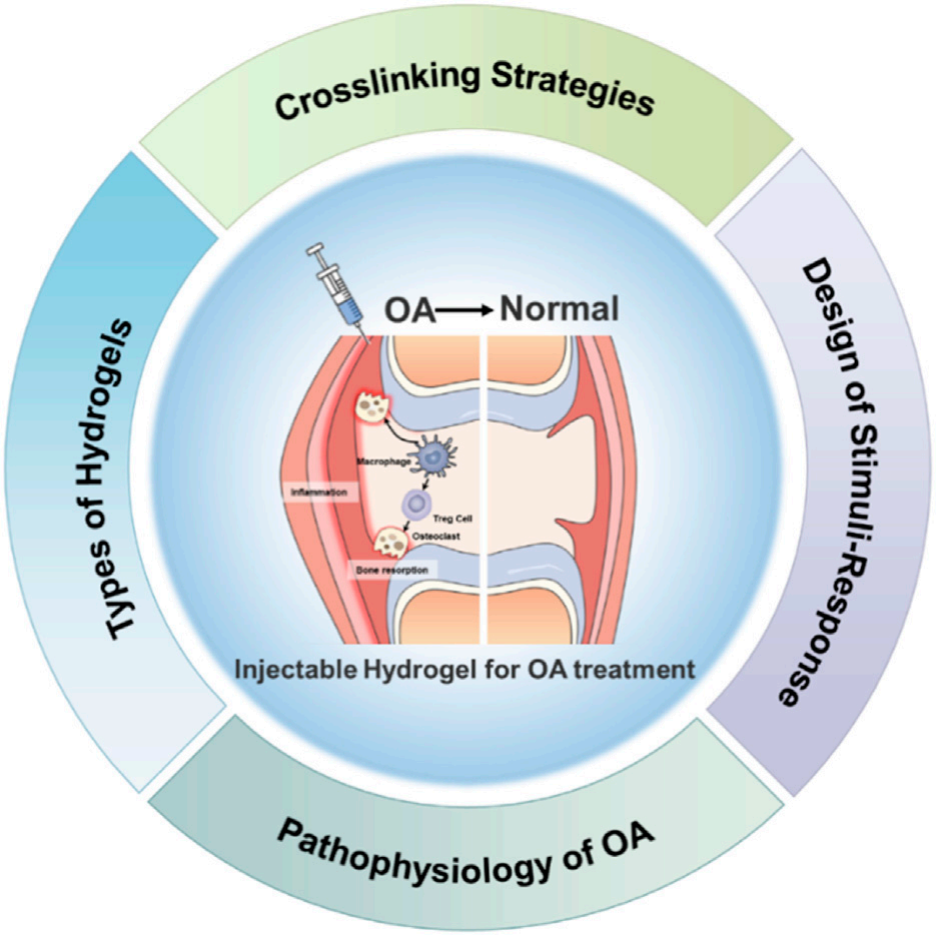
Overview of design strategies of Injectable hydrogels for OA treatment.

This review focuses on recent advances in injectable hydrogels for OA treatment, emphasizing their structural design, functional properties, and therapeutic applications (Figure 1). We discuss the underlying mechanisms of OA, highlighting key pathological features such as cartilage degradation, inflammatory responses, and subchondral bone changes. Additionally, we explore the advantages and limitations of different types of hydrogels, including natural, synthetic, and composite materials. Finally, we examine innovative cross-linking strategies and their implications for developing more effective hydrogel-based therapies. Through comprehensive analysis, this review aims to provide insights into the future directions of hydrogel research for OA treatment, paving the way for novel interventions that could significantly improve patient outcomes and quality of life.

## 2 Pathophysiology of OA

### 2.1 Cartilage degradation

Cartilage degradation is one of the most prominent pathological features of OA. This process is complex and involves abnormal changes in multiple cellular and molecular mechanisms, severely affecting joint function. Articular cartilage, a crucial component of the joint structure, is mainly composed of chondrocytes and the extracellular matrix (ECM) (Zheng et al., 2023; Zhu et al., 2023). The main components of the ECM include type II collagen, proteoglycans, and a small amount of non-collagenous proteins. Under normal physiological conditions, chondrocytes can maintain the dynamic balance between ECM synthesis and degradation, enabling normal joint movement and function. However, in the pathological process of OA, the homeostatic balance of cartilage is disrupted. The catabolic process is significantly enhanced, while the anabolic process gradually weakens (Zeng et al., 2022). The matrix metalloproteinases (MMPs) and a disintegrin and metalloproteinase with thrombospondin motifs (ADAMTS) families play key roles in cartilage degradation (Zhang et al., 2023). When the joint suffers mechanical damage, MMP-13 and ADAMTS-5 are highly expressed. MMP-13 can specifically recognize and cleave the helical structure of type II collagen, breaking it down into small-molecule fragments. This disrupts the fibrous network structure of cartilage, leading to a decline in its mechanical properties. ADAMTS-5 mainly acts on aggrecan in proteoglycans, causing a large amount of proteoglycans to be lost from the ECM (Stack and McCarthy, 2016). The reduction of proteoglycans decreases the hydrophilicity of cartilage, making it unable to store water effectively and weakening its compressive resistance. As cartilage degradation progresses, the cartilage surface gradually shows fibrosis and cracks, and eventually, the full-thickness cartilage is damaged, exposing the underlying subchondral bone, which seriously affects the normal function of the joint (Ouyang et al., 2023).

In addition to the effects of MMPs and the ADAMTS family, the abnormal behavior of chondrocytes in the pathological environment of OA also accelerates the process of cartilage degradation (Li et al., 2021). In the inflammatory microenvironment, the anabolic function of chondrocytes is inhibited. Their ability to synthesize ECM components such as type II collagen and proteoglycans is significantly reduced, and they cannot replenish the degraded matrix components in a timely manner. At the same time, chondrocytes in the OA state are more prone to senescence and apoptosis. Senescent chondrocytes secrete a series of senescence-associated secretory phenotype (SASP) factors, which include various pro-inflammatory factors and catabolic enzymes, further exacerbating the inflammatory response and cartilage degradation (Lafont et al., 2023). The apoptosis of chondrocytes directly reduces the number of cells that can maintain the structure and function of cartilage, greatly weakening the self-repair ability of cartilage (Ibáñez et al., 2022).

Furthermore, abnormal changes in subchondral bone are closely related to cartilage degradation. During the development of OA, subchondral bone shows abnormal bone remodeling, manifested as osteosclerosis and changes in trabecular bone structure (Fang et al., 2021). These changes in bone structure lead to alterations in the joint mechanical environment, causing uneven stress distribution on the cartilage and further aggravating cartilage damage and degradation. Meanwhile, cytokines and growth factors released from subchondral bone can diffuse into cartilage tissue, affecting the function and metabolism of chondrocytes and promoting the process of cartilage degradation (Cho et al., 2021; Duan et al., 2020).

### 2.2 Inflammatory response

The inflammatory response is another crucial factor in the pathological process of OA. Synovitis not only exacerbates the inflammatory environment within the joint but also promotes further damage to cartilage and bone tissues. Research shows that approximately 50%–80% of OA patients develop synovitis (Syed et al., 2024). The presence of synovitis significantly deteriorates the microenvironment within the joint, leading to a large accumulation of inflammatory factors and further aggravating the damage to cartilage and bone tissues, creating a vicious cycle (Sewell and and Östör, 2022). Interleukin-1β (IL-1β) and tumor necrosis factor-α (TNF-α) are key pro-inflammatory cytokines in the OA inflammatory response (Roseti et al., 2019). In OA joints, multiple stimulatory factors can prompt synovial cells, macrophages, and chondrocytes to secrete IL-1β and TNF-α. IL-1β can upregulate the expression of various catabolic enzymes, such as matrix metalloproteinases (MMPs) and members of the a disintegrin and metalloproteinase with thrombospondin motifs (ADAMTS) family, by activating the nuclear factor-κB (NF-κB) signaling pathway, thus promoting the degradation of the cartilage matrix. Studies have shown that after IL-1β stimulates chondrocytes, the expression of MMP-13 and ADAMTS-5 increases significantly, accelerating cartilage destruction (Protano et al., 2023). At the same time, IL-1β can also inhibit the synthesis of type II collagen and proteoglycans by chondrocytes, further weakening the structure and function of cartilage. TNF-α also has a powerful pro-inflammatory effect. It can not only enhance the angiogenic activity of synovial cells, leading to synovial tissue hyperplasia and pannus formation, which in turn erodes cartilage and subchondral bone, but also synergize with IL-1β to amplify the inflammatory response and exacerbate joint damage (Nees et al., 2019).

In addition to IL-1β and TNF-α, other inflammatory mediators also play important roles in the inflammatory response of OA. Prostaglandin E2 (PGE2), an inflammatory mediator produced by the cyclooxygenase (COX) pathway, shows a significantly elevated level in OA joints (Liu et al., 2023). PGE2 inhibits the proliferation and differentiation of chondrocytes and promotes their apoptosis by activating the cyclic adenosine monophosphate (cAMP) signaling pathway. Meanwhile, PGE2 can increase vascular permeability and promote the infiltration of inflammatory cells, further aggravating joint inflammation. Moreover, PGE2 is closely related to pain generation as it can sensitize nociceptors, making patients experience more intense pain. Reactive oxygen species (ROS) also play a significant role in the OA inflammatory response. In OA joints, factors such as the activation of inflammatory cells, mitochondrial dysfunction, and metabolic abnormalities lead to an increase in ROS production (Knights et al., 2023). ROS are highly reactive and can oxidize intracellular biomacromolecules, such as lipids, proteins, and DNA, resulting in cell damage. At the same time, ROS can activate signaling pathways such as NF-κB, further promoting the expression and release of inflammatory factors and forming a vicious cycle. Studies have found that the level of ROS in the synovial fluid of OA patients is positively correlated with the severity of the disease, indicating that ROS play a key role in the inflammatory progression of OA (Di Francesco et al., 2022).

Furthermore, the inflammatory response can trigger the infiltration of immune cells and synovial hyperplasia within the joint. In the early stage of OA, the synovial tissue shows mild inflammation, manifested as the hyperplasia of synovial cells and the infiltration of inflammatory cells (Chow and Chin, 2020). As the disease progresses, the synovial inflammation gradually worsens, and the synovial tissue undergoes obvious hyperplasia and thickening, forming pannus. Pannus can invade cartilage and subchondral bone, releasing various proteases and inflammatory factors and directly destroying joint tissues. Meanwhile, the aggregation of immune cells in the joint leads to the abnormal activation of the immune response, further exacerbating the inflammatory response and forming a self-perpetuating inflammatory cycle.

### 2.3 Current treatments for OA

Despite the growing understanding of the pathological mechanisms of OA, there remains a lack of effective clinical treatments capable of reversing the disease progression. Current treatment modalities mainly encompass non-steroidal anti-inflammatory drugs (NSAIDs), glucocorticoid injections, physical therapy, and lifestyle interventions (Chalidapong et al., 2024). These are designed to alleviate symptoms and enhance the quality of life of patients. However, most of these approaches can only provide temporary pain relief and fail to halt or reverse cartilage degradation and the inflammatory response. For patients with advanced OA, joint replacement surgery represents an effective treatment option. Nevertheless, its high cost and potential complications limit its widespread application. Moreover, long-term use of NSAIDs and glucocorticoids may lead to a series of side effects (Braaten et al., 2022). For instance, NSAIDs can cause gastrointestinal ulcers, and both NSAIDs and glucocorticoids are associated with an increased cardiovascular risk (Assi et al., 2023). Additionally, glucocorticoids may contribute to further cartilage degeneration. Consequently, there is an urgent need to develop novel treatment strategies, which can slow down the disease progression, reduce side effects, and ultimately improve the overall quality of life for patients suffering from OA.

## 3 Types of injectable hydrogels for OA treatment

Against the backdrop of limitations in current OA treatments, exploring novel therapeutic avenues is crucial. The VOS Viewer map offers the research landscape of injectable hydrogels for OA treatment (Buchanan et al., 2024). The analysis data were derived from the Web of Science Core Collection (WoSCC) with the retrieval strategy of keyword combinations: (“injectable hydrogel” or “therapeutic hydrogel”) and (“osteoarthritis” or “OA”). The time range was limited to 2018–2025, and the initial search yielded 876 peer-reviewed literature. After excluding duplicate literature, non-English literature, and low-impact studies (citation count < 5), a total of 542 literature were finally included for co-occurrence analysis.

**FIGURE 2:**
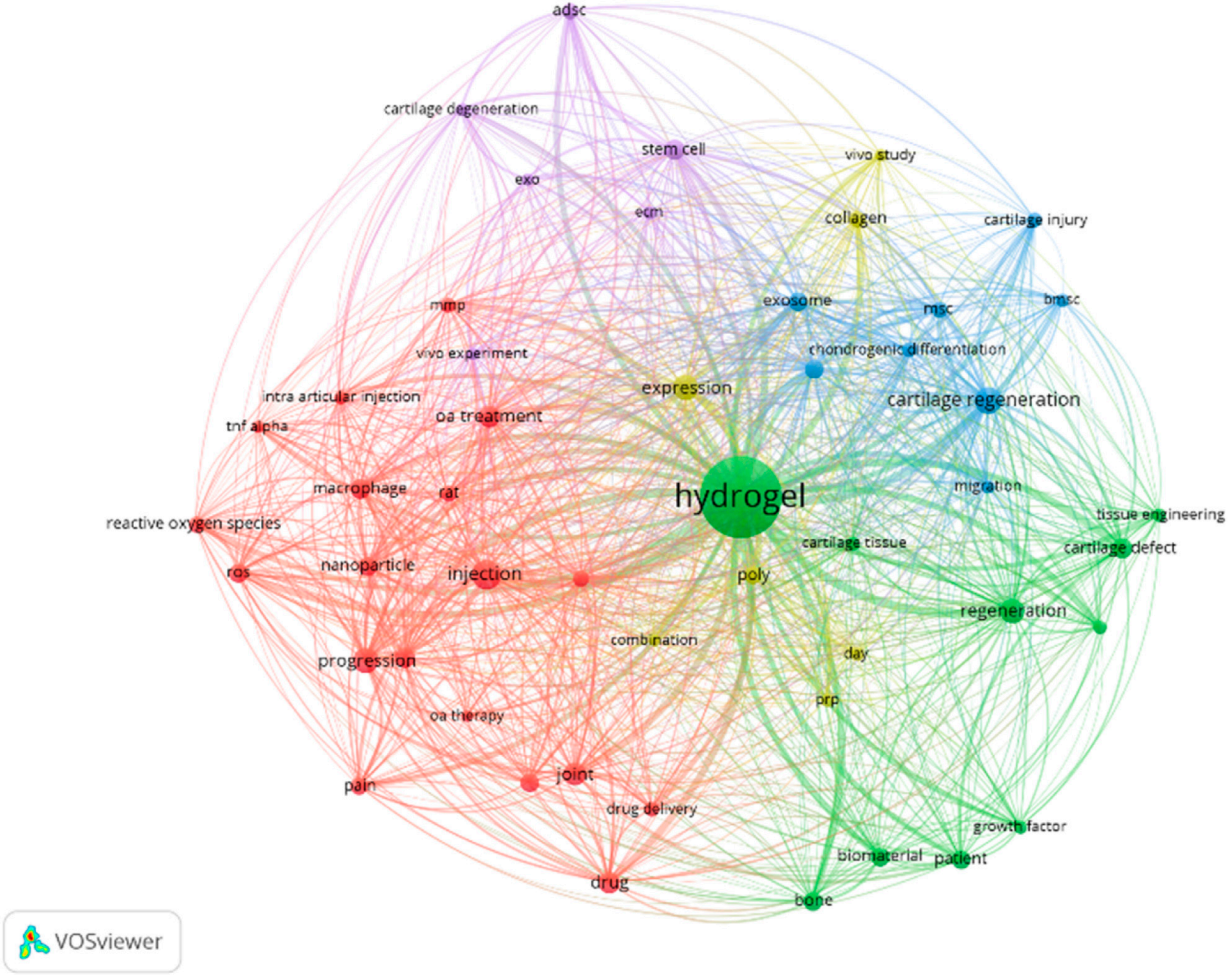
VOS viewer map of injectable hydrogels for OA treatment.

At its heart is “hydrogel,” highlighting its centrality in this research domain (Figure 2). The map organizes related terms into distinct colored clusters. The green cluster, with terms like “cartilage defect” and “tissue engineering,” showcases efforts to use hydrogels for cartilage restoration within tissue engineering contexts. The blue cluster, featuring “chondrogenic differentiation” and “exosome,” focuses on exosome-related cellular processes for cartilage repair. The red cluster, containing “OA treatment” and “intra-articular injection,” zeroes in on practical clinical applications, while the purple cluster, including “stem cell” and “cartilage degeneration,” delves into the biological underpinnings of OA. The connecting lines between terms quantify their relationships, revealing that concepts such as “drug delivery” and “biomaterial” are intricately linked to hydrogels. This indicates their promise as multifunctional agents in OA therapy.

### 3.1 Natural polymer-based hydrogels

Natural polymer-based hydrogels exhibit unique advantages in the treatment of OA due to their excellent biocompatibility, biodegradability, and similarity to the extracellular matrix. These hydrogels are mainly derived from natural biomaterials such as polysaccharides and proteins, which can provide a favorable microenvironment for cells and promote tissue repair and regeneration. HA is a crucial component of joint synovial fluid and the extracellular matrix of cartilage, plays a significant role in natural polymer-based hydrogels. Composed of D-glucuronic acid and N-acetylglucosamine linked by β-1,4 and β-1,3 glycosidic bonds, HA possesses good hydrophilicity and lubricity (Yi et al., 2022). In the treatment of OA, HA hydrogels can function in multiple ways. On one hand, they can mimic the function of joint synovial fluid, increasing joint lubrication, reducing friction, thereby alleviating joint pain and improving joint function (Zhou et al., 2024). On the other hand, HA hydrogels have certain anti-inflammatory properties, which can reduce the inflammatory response within the joint and create favorable conditions for the repair of damaged tissues.

Chitosan (CS) is another commonly used natural polymer with good biocompatibility, antibacterial properties, and low immunogenicity (Lin et al., 2024). CS can gelate under physiological conditions, and the resulting hydrogel has a certain pore structure, which is beneficial for nutrient exchange and cell adhesion and proliferation (Xuexuan et al., 2021). Pan et al. (2020) found that injectable CS thermosensitive hydrogels can provide good lubrication for the joint surface by reducing friction with surrounding tissues. At the same time, it can promote the growth and adhesion of mesenchymal stem cells (MSCs), thus protecting articular cartilage. This property makes CS hydrogels have great potential in the treatment of OA, especially in promoting cartilage repair. Alginate is a natural polysaccharide extracted from brown algae, has good biocompatibility, low toxicity, and high gelation ability (Zhao et al., 2023). Alginate hydrogels are usually prepared by ionic cross-linking and can be used as cell carriers for tissue engineering and drug delivery. In the treatment of OA, alginate hydrogels can load various bioactive substances, such as drugs and growth factors, to improve the microenvironment within the joint.

In addition to the above-mentioned common natural polymers, other natural polymers are also used to prepare hydrogels for the treatment of OA. For example, collagen, one of the main components of the extracellular matrix, has good biocompatibility and osteoinductivity. However, its weak mechanical strength often requires compounding with other polymers (Vinikoor et al., 2023). Gelatin, a hydrolysis product of collagen, has biodegradability and biocompatibility, but its mechanical properties and moisture-absorbing properties limit its single application (Li et al., 2023). Fibrinogen polymerizes to form fibrin clots during tissue injury. It can serve as a carrier for cells and drugs, promote cell adhesion and proliferation, and regulate drug release, which is helpful for improving the joint microenvironment and promoting tissue repair (Shen et al., 2025).

### 3.2 Synthetic polymer-based hydrogels

Poly(N-isopropylacrylamide) (PNIPAm) is a typical temperature-sensitive synthetic polymer. Its molecular structure contains both hydrophilic amide groups (-CONH_2_) and hydrophobic isopropyl groups (-CH(CH_3_)_2_). This unique structure endows PNIPAm with distinct temperature-responsive properties in water. At room temperature, PNIPAm exists as a homogeneous solution, facilitating injection. When the temperature rises to near body temperature, the polymer solution undergoes a phase transition and forms a three-dimensional hydrogel (Chen T. et al., 2025). This property enables *in situ* gelation *in vivo*, making it an excellent carrier for drug delivery and tissue engineering. For instance, when PNIPAm is used to prepare drug-loaded gels, it can slowly release drugs at body temperature, enabling continuous treatment of osteoarthritis (Porcello et al., 2023). However, PNIPAm has some limitations, such as poor biodegradability, low mechanical strength, and difficulties in precisely controlling drug loading and release patterns. To overcome these drawbacks, researchers often improve its performance by adding other polymers to form an interpenetrating network (IPN) and adjust its drug-loading and release behavior (Lin et al., 2022).

Polyethylene glycol (PEG) is a widely used and non-toxic synthetic polymer. It can be copolymerized with biocompatible polyesters. By regulating the composition and length of the hydrophilic PEG segments and hydrophobic polyester segments, the thermosensitivity of the hydrogel can be precisely controlled (Yang et al., 2021). For example, the BAB triblock copolymer PLGA-PEG-PLGA is formed by the ring-opening polymerization of lactide and glycolide using PEG-diol as a macro-initiator. At low temperatures, the hydrophilic PEG and hydrophobic PLGA self-assemble to form micelles. When the environmental temperature increases, the PLGA shell dehydrates and the micelles aggregate, forming a scaffold with different drug-release rates (Ding et al., 2025). This temperature-responsive drug-release property allows PEG-based hydrogels to achieve precise drug release at the site of joint inflammation, enhancing the therapeutic effect while reducing side effects on normal tissues. Polyacrylamide hydrogels are highly cross-linked three-dimensional acrylamide networks with excellent biocompatibility and water-exchange capabilities (Bliddal et al., 2024). Research shows that injectable polyacrylamide hydrogels can protect the cartilage surface and promote high-quality fibrocartilage healing, effectively alleviating the symptoms of osteoarthritis (Vishwanath et al., 2023).

Poloxamers are a class of water-soluble non-ionic triblock copolymers composed of hydrophilic polyethylene oxide and hydrophobic polypropylene oxide blocks, belonging to intelligent polymers. They are sensitive to external stimuli such as temperature, pH, and salt concentration (Mammella et al., 2024). In an aqueous solution, poloxamers form micelles with an ordered structure at appropriate temperatures and concentrations. As the temperature rises, the structural order of the micelles increases, causing them to transform from a sol state to a gel state (Jose et al., 2024). This unique property allows poloxamer hydrogels to not only be directly used as drug carriers but also to immobilize other drug-delivery carriers at the injury site, enabling controlled release of the loaded drugs.

Wang et al. (2022) developed an injectable system using a triblock synthetic hydrogel (3B-PEG IH, specifically PLCL-PEG-PLCL) loaded with neural epithelial growth factor-like protein 1 (Nell-1), and evaluated its therapeutic efficacy in temporomandibular joint osteoarthritis (TMJOA). The PLCL-PEG-PLCL hydrogel, a synthetic triblock copolymer, was physically mixed with Nell-1 rather than integrating the protein into its network structure. *In vitro* studies showed that this system regulated the expression of chondrogenesis-related genes, while *in vivo* experiments using TMJOA rabbits demonstrated its ability to prevent subchondral trabecular bone destruction, repair the proliferative and fibrous layers of the joint surface, and reduce uneven hyperplasia of the fibrocartilage layer. Among different formulations, the 20 wt% PLCL-PEG-PLCL hydrogel was identified as optimal. This study suggests that the synthetic PLCL-PEG-PLCL hydrogel, when combined with Nell-1, may reverse TMJOA-induced osteochondral degeneration via the Nfatc1-Runx3 signaling pathway, offering a promising minimally invasive approach for clinical TMJOA treatment.

### 3.3 Composite hydrogels

Composite hydrogels combine the biocompatibility of natural hydrogels with the excellent mechanical properties of synthetic hydrogels, presenting unique advantages in the treatment of osteoarthritis. By combining natural and synthetic polymers, these hydrogels can integrate the merits of both and compensate for the shortcomings of single hydrogels, offering more effective solutions for osteoarthritis treatment (Xu et al., 2025a). Li et al. (2024) constructed a composite hydrogel scaffold by combining sodium alginate (SA) and HA, and combined extracellular vesicles derived from mesenchymal stem cells (MSCs) with icariin to form a combined therapy. This composite hydrogel not only has good biocompatibility but also synergistically promotes the proliferation and migration of MSCs and chondrocytes, inhibits the inflammatory response, stimulates the synthesis of cartilage matrix components, activates the chondrogenic ability of chondrocytes, and simultaneously inhibits cartilage degradation, thus effectively promoting the repair of articular cartilage defects. In this composite system, sodium alginate provides good gelation ability and cell adhesion sites, while hyaluronic acid enhances the lubricity and bioactivity of the hydrogel. The combination of the two creates an ideal microenvironment for cell growth and tissue repair.

Long et al. (2025) developed an injectable nanocomposite hydrogel system for miRNA-based cartilage repair in knee osteoarthritis, addressing the limitations of free miR-455-3p (rapid degradation, poor targeting, and off-target effects). The system comprises a cartilage affinity nanocarrier (CANC) encapsulated in a PCL-b-PEG-b-PCL hydrogel: the CANC uses 50% PEGylated G5 PAMAM dendrimers to load miR-455-3p, with chondrocyte-affinity peptides (CAP) and minimal “self” peptides (MSP) modifying to enhance cartilage targeting and evade macrophage uptake. *In vitro* studies confirmed its excellent stability, low cytotoxicity, superior cartilage penetration, and chondrocyte targeting. *In vivo* experiments in miR-455-3p knockout mice and a destabilization of the medial meniscus (DMM)-induced KOA model demonstrated that sustained, targeted delivery of miR-455-3p effectively rescued cartilage degeneration and prevented KOA progression, with high biocompatibility and no observed cytotoxicity in major organs.

In addition, Rui et al. (2023) prepared an *in situ* photocrosslinked silk fibroin hydrogel encapsulating olfactory ectomesenchymal stem cell-derived exosomes (Exos@SFMA). This hydrogel has excellent biocompatibility and flexible mechanical properties, which can effectively protect the surface of joint tissues. In a collagen-induced arthritis (CIA) mouse model, implanting this hydrogel can significantly reduce the follicular helper cell response and further inhibit the differentiation of B cells into plasma cells, thus effectively alleviating synovitis and joint degeneration. This study demonstrates that this silk fibroin hydrogel combined with exosomes provides a powerful treatment option for the treatment of OA.

### 3.4 Structural features and therapeutic efficacy

Injectable hydrogels’ therapeutic performance in OA is profoundly influenced by their structural characteristics, particularly internal pore size and network interconnectedness. Pore size determines the capacity for drug loading and cell infiltration: Hydrogels with micron-scale pores (10–100 μm) facilitate the encapsulation of large biomolecules (e.g., growth factors) and promote chondrocyte or stem cell adhesion, supporting cartilage regeneration (Lin et al., 2025). For example, alginate hydrogels with 50 μm pores show enhanced MSC infiltration and chondrogenic differentiation compared to smaller-pored counterparts (Macdougall and Anseth, 2020). In contrast, nanoporous structures (<10 μm) are optimal for small-molecule drug delivery, preventing premature leakage while enabling controlled diffusion. Network interconnectedness (the degree of cross-linking and pore connectivity) governs mechanical stability and mass transport: Highly interconnected networks ensure efficient nutrient/waste exchange, critical for maintaining cell viability in regenerative hydrogels (Majidi et al., 2018). A study on HA-based hydrogels found that increased network connectivity (via optimized cross-linking) improved both drug release uniformity and the ability to support chondrocyte metabolism (Wang S.-J. et al., 2024). Conversely, poorly interconnected networks may lead to uneven drug distribution and limited cell survival, compromising therapeutic outcomes. These structural features are tightly linked to hydrogel functionality: For instance, a composite hydrogel with tailored pore size (30–50 μm) and high interconnectivity showed enhanced encapsulation of exosomes, prolonged release, and superior cartilage repair in OA models (Xia et al., 2022). Thus, optimizing pore size and network structure is essential for maximizing the therapeutic potential of injectable hydrogels in OA.

## 4 Design of structure and functions of injectable hydrogel for OA treatment

### 4.1 Cross-linking strategies

Selecting an appropriate cross-linking strategy is crucial for optimizing the mechanical properties, biocompatibility, and therapeutic efficacy of injectable hydrogels for osteoarthritis treatment. Different strategies offer distinct advantages and limitations, impacting their suitability for various clinical applications (Zhang et al., 2023). Table 1 provides a detailed comparison of chemical, physical, and multiple cross-linking methods, highlighting their representative examples, benefits, drawbacks, and suitable applications.

**TABLE 1:** Comparison of different cross-linking strategies for injectable hydrogels in OA treatment.

Cross-linking strategy	Representative examples	Advantages	Disadvantages	Suitable applications
Chemical Cross-linking	Glutaraldehyde, EDC/NHS	Provides highly stable three-dimensional network structures. Enables precise control over degradation rates. Suitable for long-term drug release applications.	Potential cytotoxicity; Complex preparation processes may affect biocompatibility; Not conducive to rapid molding.	Scenarios requiring high stability or specific degradation rates.
Physical Cross-linking	Hydrogen bonding, Ionic bonds	Mild conditions without the need for harmful chemicals; Easy preparation, suitable for encapsulating sensitive biomolecules or cells.	Lower mechanical strength; Poor structural stability may lead to premature drug leakage.	Situations involving live cells or temperature-sensitive biological molecules.
Multiple Cross-linking Methods	Combination of above methods	Integrates advantages of both chemical and physical cross-linking, offering high flexibility; Enhanced mechanical properties and controllable degradation behaviors; Improved biocompatibility.	Relatively complex preparation techniques; Requires precise regulation of component ratios to achieve optimal results.	Applications demanding higher mechanical performance or multifunctional responsive features.

#### 4.1.1 Chemical cross-linking

Chemical cross-linking is a method that uses chemical reagents or initiators to form a stable three-dimensional network structure, thereby enhancing the mechanical strength and stability of hydrogels. This approach usually involves the formation of covalent bonds, which can significantly improve the durability of hydrogels and their ability to control drug release. Commonly used chemical cross-linkers include glutaraldehyde, EDC/NHS, and genipin. Glutaraldehyde is a classic cross-linker and is often used for the cross-linking of natural polymers such as gelatin and chitosan. However, glutaraldehyde has certain cytotoxicity, which limits its wide application in the biomedical field. Genipin, a natural cross-linker derived from gardenia fruit, offers a more biocompatible alternative. It effectively cross-links polymers like chitosan and gelatin with minimal cytotoxicity, making it suitable for biomedical applications where biocompatibility is critical (Xu D. et al., 2025). In contrast, the EDC/NHS system is milder. It can achieve the cross-linking of proteins or polypeptides through the formation of amide bonds and has relatively low cytotoxicity. For example, hydrogels containing hyaluronic acid and EDC/NHS cross-linkers have been proven to effectively promote cartilage repair and reduce the inflammatory response (Zhang et al., 2023). Moreover, this hydrogel also exhibits good *in vivo* degradation properties, gradually degrading and being absorbed by the body within a few weeks, avoiding potential risks associated with long-term implants.

Notably, dynamic covalent chemistry has emerged as a promising cross-linking strategy in recent years, bridging the gap between traditional chemical and physical cross-linking (Zhou et al., 2022). This approach utilizes reversible covalent bonds (e.g., imine bonds, disulfide bonds, and boronate esters) that can undergo bond cleavage and reformation under physiological conditions. These dynamic bonds enable hydrogels to remodel their network structure in response to external stimuli (such as pH or redox changes) – a characteristic similar to physically cross-linked gels–while maintaining higher structural stability compared to purely physical networks, akin to conventional chemical cross-linking (Lei et al., 2022). For instance, hydrogels cross-linked via imine bonds (formed between aldehyde and amine groups) can dynamically adjust their mechanical properties in the acidic OA microenvironment, facilitating controlled drug release and adapting to joint movement (Zhang B. et al., 2025). This unique combination of stability and responsiveness makes dynamic covalent cross-linking particularly attractive for OA treatment, where the hydrogel needs to withstand mechanical stress while adapting to the pathological microenvironment.

The main advantage of chemical cross-linking is its ability to provide a highly controllable cross-linking density and network structure, enabling precise regulation of the mechanical properties and degradation rate of hydrogels. Additionally, chemical cross-linking can introduce functional groups, such as bioactive peptides, growth factors, or other bioactive molecules, further enhancing the therapeutic efficacy of hydrogels. For instance, a chemically cross-linked hydrogel based on hyaluronic acid and methacrylate was designed for the delivery of mesenchymal stem cells (MSCs). By adjusting the cross-linking density, effective control over the proliferation and differentiation of MSCs was achieved. Another common chemical cross-linking method is cross-linking under ultraviolet or visible light irradiation using photoinitiators. For example, a methacrylate-modified hyaluronic acid (HA-MA) hydrogel can be rapidly cross-linked under blue-light irradiation with the help of a photoinitiator. This hydrogel has excellent injectability and shape adaptability, allowing it to closely conform to irregular joint surfaces. In an OA rat model, this photocross-linked hydrogel significantly improved the repair effect of articular cartilage and reduced pain symptoms (Yang et al., 2020).

However, during the chemical cross-linking process, unreacted cross-linkers may remain, leading to potential cytotoxicity issues. Therefore, the selection of low-toxicity cross-linkers and the optimization of cross-linking conditions are of great importance. Secondly, it is difficult to precisely control the degradation rate of chemically cross-linked hydrogels, which may result in problems such as premature degradation or long-term retention. To address this, researchers are exploring the combination of multiple cross-linking mechanisms to achieve more flexible degradation regulation.

#### 4.1.2 Physical cross-linking

Physical cross-linking refers to the formation of a three-dimensional network structure through non-covalent bonds (such as hydrogen bonds, ionic bonds, van der Waals forces, etc.) or physical effects (such as temperature changes, light irradiation, etc.). Common physical cross-linking methods include temperature-sensitive cross-linking and ionic cross-linking. Among them, temperature-sensitive cross-linking is based on the phase separation phenomenon of polymer solutions caused by temperature changes, enabling the formation of hydrogels. For example, polyethylene glycol-polylactic acid (PEG-PLA) block copolymers are in a liquid state at low temperatures and rapidly transform into a gel state at body temperature. This property makes it an ideal injectable hydrogel material. Research shows that this thermosensitive hydrogel can effectively deliver drugs and slowly release them in the local environment, thus prolonging the therapeutic effect (Choi et al., 2025). Ionic cross-linking is another commonly used physical cross-linking method, which mainly relies on the electrostatic interaction between charged polymers to form a stable network structure. For instance, chitosan, a cationic polysaccharide, can undergo ionic cross-linking with anionic polysaccharides such as hyaluronic acid or chondroitin sulfate to form hydrogels with excellent biocompatibility (Bandyopadhyay et al., 2024).

Although physical cross-linking technology shows great potential in hydrogel design, physically cross-linked hydrogels usually have relatively weak mechanical properties. Secondly, it is difficult to precisely control the degradation rate of physically cross-linked hydrogels, which may lead to problems such as premature degradation or long-term retention. Therefore, researchers need to further optimize the cross-linking conditions and material selection to achieve more flexible degradation regulation.

#### 4.1.3 Multiple cross-linking

The multiple cross-linking strategy is increasingly attracting extensive attention from researchers in the field of injectable hydrogels for the treatment of OA. Compared with single cross-linking methods, multiple cross-linking endows hydrogels with more excellent comprehensive properties by combining the stability of chemical cross-linking and the dynamics of physical cross-linking. These properties include enhanced mechanical strength, controllable degradation rate, and adjustable drug-release behavior. The strategy of combining chemical and physical cross-linking is one of the most representative multiple cross-linking methods. This approach fully utilizes the permanent connections provided by chemical cross-linking to ensure the stability of the basic framework of the hydrogel. Meanwhile, physical cross-linking imparts the hydrogel with dynamically adjustable properties, such as responsiveness to temperature, pH, or specific biomolecules. For example, researchers have successfully constructed a multiple-cross-linked hydrogel with both chemical stability and temperature responsiveness by chemically cross-linking chitosan and hyaluronic acid while introducing temperature-sensitive polyethylene glycol (PEG) segments (Mou et al., 2021). At body temperature, the PEG segments can form hydrogen bonds with chitosan and hyaluronic acid, further enhancing the mechanical strength of the hydrogel. When the hydrogel is implanted into the joint microenvironment, with the local temperature changes caused by the inflammatory response, the PEG segments can dynamically dissociate from the hydrogel, releasing the encapsulated therapeutic drugs and achieving precise drug release.

In addition, using the cross-linking effect of biomolecules is also an important strategy for achieving multiple cross-linking. This method can not only reduce the potential biological toxicity of chemical cross-linkers but also enable the hydrogel to better integrate into the biological environment. For instance, researchers have utilized the extracellular matrix properties of fibronectin (FN) and introduced it as a biological cross-linker into the hydrogel system (Wei et al., 2022). FN can interact with the polymer chains in the hydrogel through its multiple domains, forming physical cross-linking points. At the same time, researchers have also covalently linked FN to other components in the hydrogel through chemical methods, forming a network structure with both chemical and physical cross-links. After being implanted into the body, this hydrogel can interact well with the surrounding tissues, improving biocompatibility and therapeutic effects. Besides combining chemical and physical cross-linking, combining multiple physical cross-linking methods is also an effective way to achieve multiple cross-linking. For example, researchers have introduced temperature-and pH-sensitive polymer segments into the hydrogel system (Seo et al., 2022). Under different environmental conditions, these polymer segments can form different interactions, such as hydrogen bonds, hydrophobic interactions, and electrostatic interactions, making the hydrogel multi-responsive. After the hydrogel is implanted into the body, its mechanical properties and drug-release behavior can be dynamically adjusted according to changes in the local environment to meet the needs of different treatment stages.

When designing multiple-cross-linked hydrogels, multiple factors need to be comprehensively considered, such as cross-linking density, the types and degrees of cross-linking methods, the length and properties of polymer segments, etc., to ensure that the hydrogels have ideal properties. Moreover, researchers can also control the distribution and density of cross-linking points to regulate the degradation rate and drug-release behavior of hydrogels, achieving sustained-release or controlled-release effects of drugs. However, the clinical application of multiple-cross-linked hydrogels still faces many challenges. The degradation and drug-release behavior of hydrogels in the body may be affected by various factors, such as changes in the local microenvironment and the action of enzymes. Therefore, it is necessary to conduct in-depth research on their degradation mechanisms and drug-release laws. In addition, the preparation methods of multiple-cross-linked hydrogels are complex, and it is difficult to achieve large-scale production, which also limits the promotion of their clinical applications.

### 4.2 Stimuli-responsive injectable hydrogels

The complex pathological microenvironment of OA, characterized by elevated temperature, acidic pH, and abnormal enzyme activity, necessitates the development of intelligent hydrogels capable of responding to multiple stimuli. Stimuli-responsive injectable hydrogels have emerged as a promising strategy, enabling precise drug delivery and localized therapy by adapting to OA-specific cues. These hydrogels can be designed to respond to thermal, pH, enzymatic, or combined stimuli, addressing key challenges such as uncontrolled drug release and poor specificity. Below is a summary of representative examples of stimuli-responsive hydrogels, highlighting their material composition, functional design, and therapeutic outcomes in preclinical OA models (Table 2).

**TABLE 2:** Summary of applications of stimuli-responsive injectable hydrogels for OA treatment.

Hydrogel material	Loaded drug/Functional material	Crosslinking strategy	Design strategies	Experimental results	Reference
HA, polyethylene glycol (PEG), mesoporous polydopamine nanoparticles (MPDANPs)	Methotrexate (MTX)	Chemical cross-linking (Nb and Tz-based “bio-orthogonal chemistry” reaction)	ROS-responsive (scavenged ROS and self-regulated MTX release)	Hydrogel relieved inflammation, prevented bone erosion in CIA rats.	Xu et al. (2024)
Pluronic^®^ F-127, HA	β-Lapachone	Physical cross-linking (self-assembly of Pluronic^®^ F-127 micelles and interaction with HA)	Thermosensitive (sol-gel transition at body temperature)	Hydrogel reduced inflammation, improved cartilage and bone histomorphometric markers.	Rouco et al. (2025)
Methylcellulose (MC), HA, methionine-modified carboxymethyl chitosan (CCM), ZIF-8	Quercetin (Que)	Physical cross-linking (self-assembly of MC and hydrogen bonding with CCM@ZIF-8@Que)	Thermosensitive, pH and ROS-responsive	Hydrogel scavenged ROS, promoted cartilage repair in OA rats.	Zhang et al. (2025b)
Poly(N-acryloyl alaninamide) (PNAAA), platelet lysate (PL)	platelet lysate	Physical cross-linking (hydrogen-bond crosslinking network formed by mixing PNAAA powder and PL solution)	Thermosensitive	Attenuated cartilage degeneration and synovitis in rat OA model.	Zhang et al. (2024a)
Phenylboronic acid-modified gelatin methacryloyl (GP)	epigallocatechin-3-gallate (EGCG)	Chemical cross-linking (formation of boronic esters between GP and EGCG)	ROS-and pH-responsive (responsive release of EGCG under high ROS levels and acidic conditions)	Ameliorated intervertebral disc degeneration in rat model.	Liu et al. (2024a)
Chitosan (CS), sodium beta-glycerophosphate (β-GP), HA	melittin (MLT)	Chemical cross-linking (using EDC/NHS to activate carboxyl groups of HA and incorporate it into CS/GP-Gel)	pH-responsive (acid-responsive hydrogel for targeted drug release in OA acidic microenvironment)	Restored Th17/Treg-mediated immunity balance, reduced inflammatory factor release, alleviated inflammation in mouse OA model.	Liu et al. (2024b)
N-(2-hydroxypropyl) methacrylamide (HPMA) copolymer	Hydromorphone (HMP)	Physical cross-linking (self-assembly driven by high HMP base content at 37°C)	Thermosensitive (sol-gel transition at 37°C for local retention)	Retained in mouse knee joints for at least 2 weeks post-injection with low extra-articular distribution; provided sustained joint pain relief for >14 days without systemic analgesia or tolerance in DMM mouse model.	Jia et al. (2024a)
Hyaluronic acid	Celecoxib (CLX)-loaded HSPC liposomes	Dynamic covalent bonds (Schiff base reaction between HA-CHO and HA-ADH)	Shear-responsive boundary lubrication	Maintained stable lubrication (coefficient of friction = 0.031); alleviated cartilage wear; regulated ECM anabolic-catabolic balance; attenuated OA progression in rat models	Lei et al. (2022)
Oxidized hyaluronic acid (OHA)/quaternized chitosan (QCS)	Diacerein (DIA) microspheres; nano-hydroxyapatite (nHAP)	Dynamic crosslinking (Schiff base reaction between OHA and QCS; hydrazide bonds with ADH; hydrogen bonds with PVA)	pH-responsive degradation and drug release	Extended drug release to 47 days; pH-dependent degradation; promoted cartilage repair in rabbit OA models; sustained release in joint cavity for over 31 days	Zhou et al. (2022)
Hyaluronic acid/platelet-rich plasma (PRP)	BSA-MnO_2_ (BM) nanozymes; growth factors in PRP	Schiff base reaction (between aldehyde-modified HA and adipic dihydrazide-grafted HA)	pH-responsive release of BM NPs and growth factors	Scavenged over 90% of ROS; enhanced chondrocyte proliferation; reduced cartilage matrix degradation; improved OA symptoms in rat models (lower Mankin score)	Zhang et al. (2025a)
Liposome/siMMP13, NG-Monomethyl-L-arginine Acetate (L-NMMA), Pluronic F-127, hyaluronic acid modified with phenylboronic acid (HA-PBA)	siMMP13, L-NMMA	Physical cross-linking (thermosensitive F127/HAPBA hydrogel)	RNA interference-based (targets MMP13), anti-ROS, anti-apoptotic	Delayed OA progression, preserved cartilage integrity in DMM model mice.	Ji et al. (2024)
cartilage decellularized matrix (DECM)	Protocatechualdehyde (PAH)	Chemical cross-linking (Schiff base reaction between PAH and DECM)	Targeted therapy (targets EPYC), sustained release (delays PAH release)	Alleviated OA symptoms, reduced cartilage loss in rat model.	Huang et al. (2024)
HA, collagen, bisphosphonate-functionalized HA macromers (HABP)	Zn-doped biphasic calcium phosphate (ZnBCP)	Chemical cross-linking (bio-orthogonal reaction between HA and collagen, electrostatic interaction between HABP and ZnBCP)	Osteo-immunomodulation (modulates M2 macrophages and osteoclasts), osteogenic promotion	Enhanced bone regeneration in collagen-induced arthritis rabbit model.	Liu et al. (2024c)
6-O-stearoyl-L-ascorbic acid (SAA)	9-aminoacridine (9AA)	Physical cross-linking (heat-cool method)	Enzyme-responsive (responsive to esterase and MMPs), anti-inflammatory, cartilage-repairing	Alleviated paw swelling, restored cartilage in collagen-induced arthritis rat model.	Ali et al. (2024)
Gelatin, polyethylene glycol diacrylate (PEGDIA)	leonurine (Leon)	Physical cross-linking (phase-separation of gelatin and PEGDIA chains)	M1 macrophage-targeting, anti-inflammatory, chondroprotective	Reduced joint swelling and inflammation, protected cartilage in collagen-induced arthritis rat model.	Yang et al. (2024)
Silk fibroin (SF), oxidized pullulan (oxPL)	platinum (Pt) nanozyme	Chemical cross-linking (Schiff-base reaction between SF and oxPL)	ROS-scavenging, anti-ferroptosis, cartilage-protective	Protected and regenerated cartilage, reduced inflammation in anterior cruciate ligament transection-induced OA rat model.	Feng et al. (2024)
HA, Polyvinyl Alcohol (PVA)	rutin, black phosphorus nanosheets (BP)	Physical cross-linking (mixing of components)	Photothermal-responsive (under NIR irradiation), anti-inflammatory	Reduced joint swelling, inflammation in OA OAt model.	Hou et al. (2024)
HA, methoxy polyethylene glycol-b-poly(ε-caprolactone)-ran-poly(lactide) (PC)	cyclic phage-display-derived inhibitory peptide (CP)	Chemical cross-linking (CP conjugated to HA) for PC+(HA-CP); Physical mixing for PC+(HA + CP)	Sustained-release depot (injectable hydrogel for local drug delivery), anti-inflammatory (inhibits TLR4 signaling)	Prolonged CP retention in joint, reduced OA symptoms in OAt model.	Lee et al. (2024)
Type II collagen	ferrous/ferric ions	Dynamic cross-linking (coordination between ferrous/ferric ions and type II collagen)	Self-healing, injectable, anti-inflammatory, chondrogenic differentiation promotion	Accelerated cartilage regeneration in rat cartilage defect model.	Wang et al. (2024b)
Aldehyde-and methacrylic anhydride-modified hyaluronic acid (AH), extracellular matrix (ECM) derived from IFN-γ-stimulated mesenchymal stem cells (MSCs)	Bioactive factors in ECM	Chemical cross-linking (photopolymerization of modified HA)	Targeted adhesion (to damaged cartilage), stem cell recruitment and differentiation promotion	Improved joint space width, cartilage integrity in rat OA model.	Pang et al. (2024)

#### 4.2.1 Thermosensitive hydrogels

The temperature at the joints of OA patients is usually elevated, which provides an opportunity for the application of thermosensitive hydrogels in OA treatment. The main mechanism of action of thermosensitive hydrogels is based on their thermosensitive groups. When the environmental temperature reaches the gelation temperature, the hydrogels can rapidly undergo a phase transition from a hydrophilic state to a hydrophobic state, thereby enabling controlled drug release (Li et al., 2018). An ideal thermosensitive hydrogel should be in a flowing state at room temperature for easy injection and quickly transform into a non-flowing gel state at physiological temperature (32°C–37°C) (Xu et al., 2025c).

**FIGURE 3:**
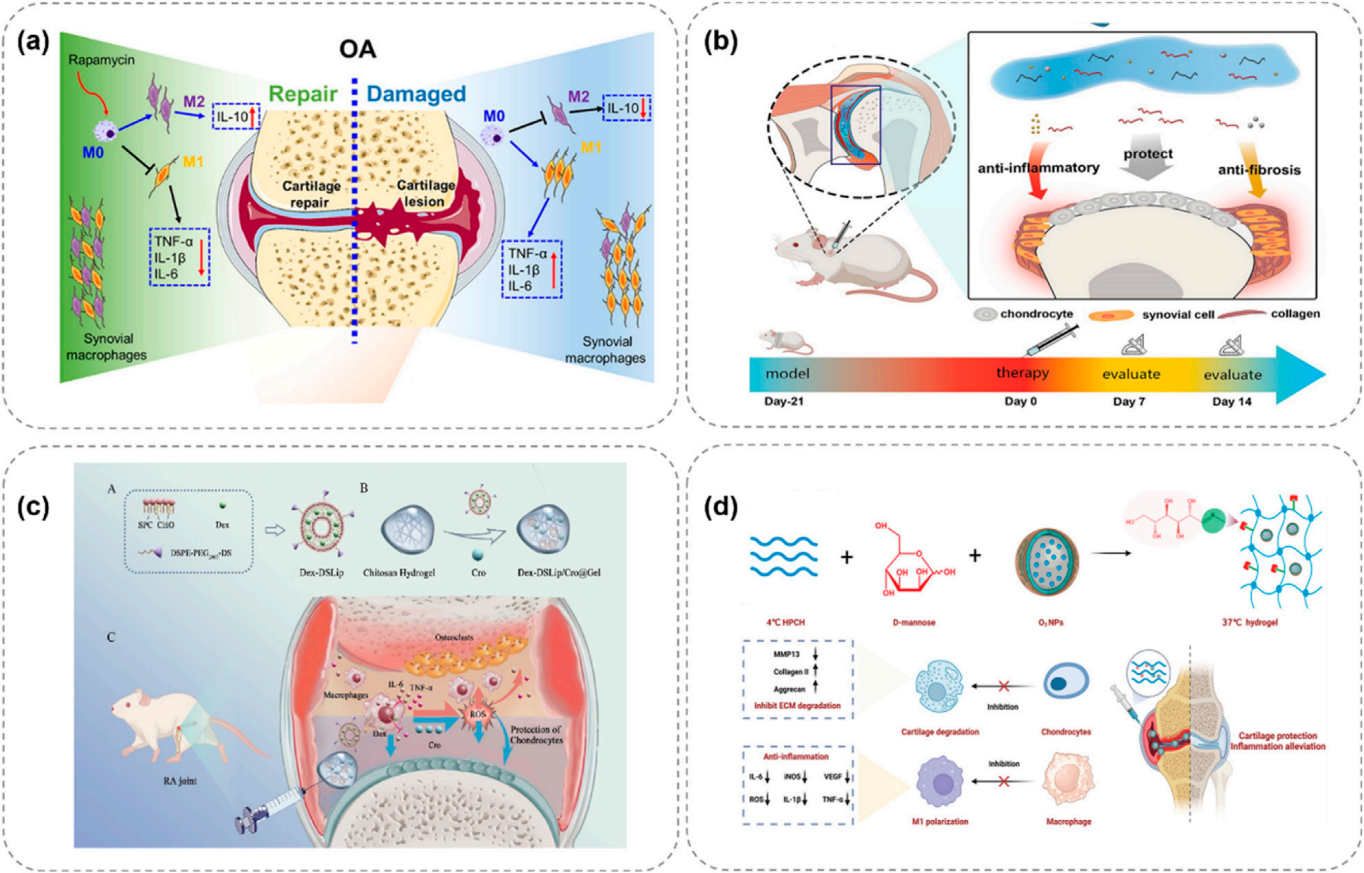
Schematic illustrations of thermosensitive hydrogel systems for treating OA through controlled release of therapeutic agents. **(a)** P-HA releases rapamycin at 37°C, polarizing macrophages to reduce OA inflammation (Chen et al., 2023). **(b)** Pluronic F-127/HA delivers drugs, easing OA via anti-inflammation (Zhang J. et al., 2024). **(c)** Chitosan gel with Dex-DSLip/Cro targets RA macrophages, lowering ROS (Liu H. et al., 2024). **(d)** Ozone-rich gel releases D-mannose, inhibiting MMP13 to protect OA cartilage (Wu H. et al., 2024).

Chen et al. (2023) constructed an injectable thermosensitive hydrogel loaded with rapamycin (P-HA hydrogel) using poloxamer 407 and hyaluronic acid (Figure 3a). This hydrogel is in a solution state at 4°C, facilitating storage and injection. After being injected into the joint, it rapidly gels at 37°C and continuously releases rapamycin. Research has found that rapamycin can induce the polarization of macrophage phenotypes from the pro-inflammatory M1 type to the anti-inflammatory M2 type, significantly reducing the expression of pro-inflammatory cytokines (such as TNF-α, IL-1β, and IL-6) in synovial tissues and synovial fluid, while increasing the expression of the anti-inflammatory cytokine IL-10. Thus, it effectively inhibits cartilage destruction and shows great potential in OA treatment. Zhang J. et al. (2024) designed an injectable thermosensitive hydrogel composed of Pluronic F-127 and HA for loading dexamethasone to treat OA (Figure 3b). This hydrogel remains stable at ambient temperature and can rapidly transform into a gel state at body temperature, achieving highly controllable release of hydrocortisone. *In vivo* experiments have shown that this hydrogel can effectively inhibit OA synovial inflammation, slow down cartilage degradation, and improve disease prognosis, highlighting the application value of thermosensitive hydrogels in OA treatment.

Liu H. et al. (2024) developed a thermosensitive injectable hydrogel, Dex-DSLip/Cro@Gel, for rheumatoid arthritis (RA) treatment (Figure 3c). In RA, there are a large number of infiltrated immune cells and abnormally elevated reactive oxygen species (ROS) in the joint. After injection in RA rats, it significantly reduced the expression of inflammatory factors in ankle joints, alleviated joint erythema and bone erosion, demonstrating targeted anti-inflammatory and antioxidation effects. Wu H. et al. (2024) developed an ozone-rich thermosensitive nanocomposite hydrogel loaded with D-mannose for OA treatment (Figure 3d). Ozone (O3) has anti-inflammatory properties but its high reactivity and short half-life limit its application. In this hydrogel, O3 is encapsulated in nanoparticles to improve its stability, and D-mannose is conjugated to form MHPCH. *In vitro* cell experiments showed that it reduced VEGF and inflammation levels, promoted the expression of collagen II and aggrecan, and stimulated chondrocyte proliferation. *In vivo* studies also demonstrated its effectiveness in alleviating OA by reducing synovial inflammation, cartilage destruction, and subchondral bone remodeling.

Although thermosensitive hydrogels show certain advantages in OA treatment, such as controllability in response to external thermal regulation and non-invasive stimulation, they still face some challenges in practical applications. For example, in deep tissues, the thermal effect of hydrogels is limited by the heat transfer efficiency, making it difficult to precisely control temperature changes to achieve the desired drug release (Wang M. et al., 2024). In addition, individual differences in joint temperatures and the influence of external environmental temperatures may lead to unstable gelation and drug release of hydrogels, thus affecting the treatment effect (Duan et al., 2023). Therefore, in the future, it is necessary to further optimize the design of thermosensitive hydrogels to improve their stability and effectiveness in different environments, so as to better meet the clinical needs of OA treatment.

#### 4.2.2 pH-responsive hydrogels

During the development of OA, the pH of the joint microenvironment undergoes significant changes. Under normal circumstances, the pH of a healthy joint is typically stable between 7.4–7.8. However, in OA patients, due to the infiltration of inflammatory cells and alterations in cell metabolism, the joint microenvironment gradually becomes weakly acidic, with the pH dropping to 6.2–6.6 (Qin et al., 2025). The mechanism of action of pH-responsive hydrogels is mainly based on the cleavage of acid-sensitive bonds or the protonation of chemical groups.

**FIGURE 4:**
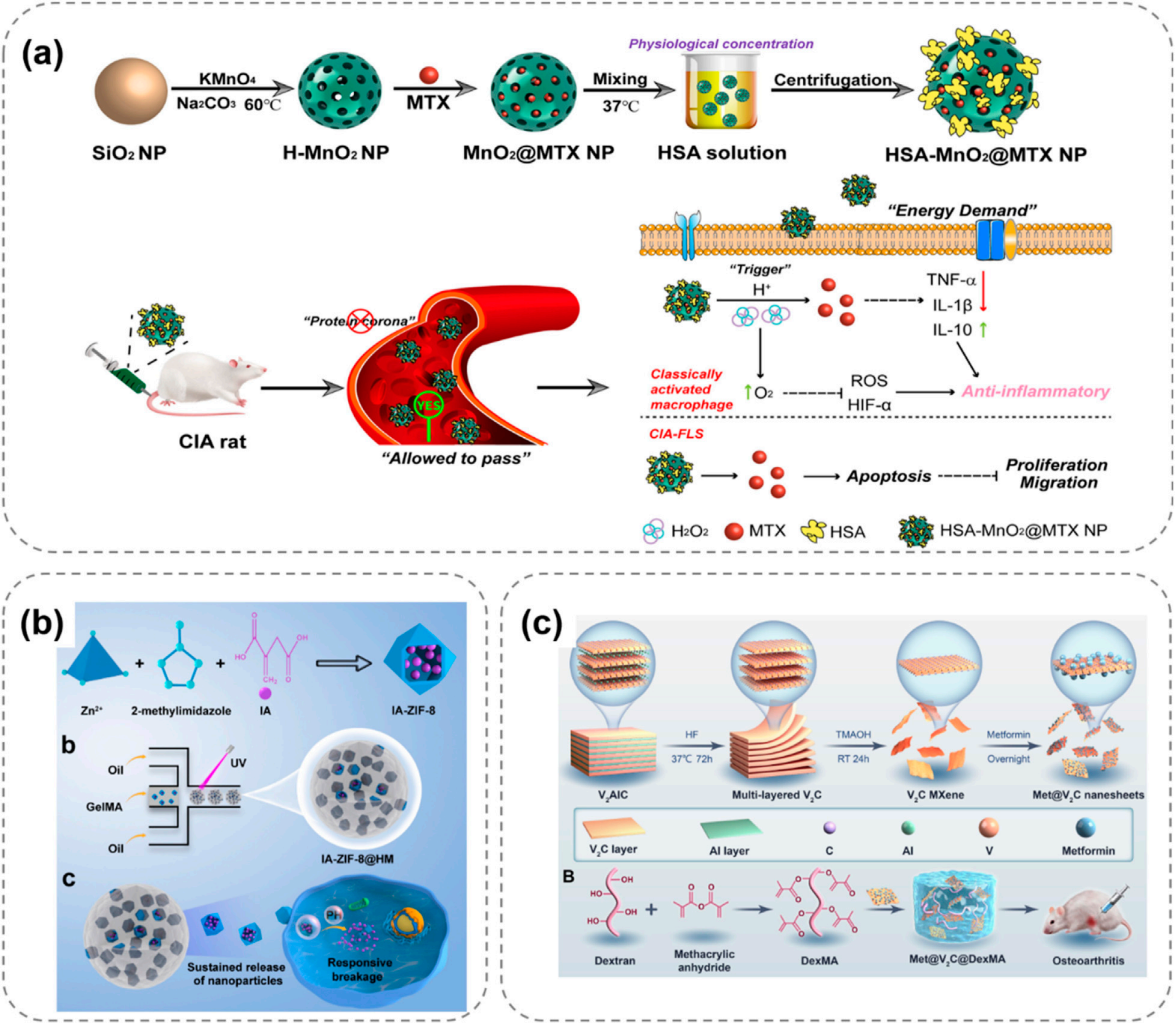
pH-responsive hydrogel systems for OA treatment, illustrating stimuli-triggered drug release and therapeutic mechanisms. **(a)** HA-PRP hydrogel releases BSA-BM nanoparticles under acidic OA conditions via Schiff base cleavage, scavenging ROS to reduce inflammation (Jia M. et al., 2024). **(b)** IA-ZIF-8@HMs microspheres respond to OA’s acidity, releasing IA to inhibit oxidative stress and inflammatory factors in chondrocytes (Yu et al., 2023). **(c)** MXenzyme-based hydrogel intelligently releases metformin in acidic environments, scavenging ROS to protect chondrocyte mitochondria (Zhang Z. et al., 2024).

In the first mechanism, researchers introduce acid-sensitive bonds into the polymer structure to serve as linkers between the drug and the carrier. When the environmental pH changes, these acid-sensitive bonds break, prompting the release of drug molecules. For example, Jia M. et al. (2024) developed a hydrogel network composed of HA and platelet-rich plasma (PRP), encapsulating bovine serum albumin (BSA) and manganese dioxide (BM) nanoparticles (Figure 4a). In this hydrogel, the active aldehyde groups on oxidized hyaluronic acid (HA-ALH) react with the amino groups on adipic dihydrazide-grafted hyaluronic acid (HA-ADH) to form Schiff bases, endowing the hydrogel with pH responsiveness. Under normal physiological conditions, the hydrogel remains stable. In an inflammatory environment (with a lower pH), the Schiff bases break, and the release rate of the hydrogel accelerates, meeting the demand-driven delivery requirements of BM nanoparticles and growth factors in the OA microenvironment. Moreover, BSA-BM nanoparticles not only possess antioxidant properties but can also rapidly scavenge various reactive oxygen species (ROS), such as superoxide anions and hydroxyl radicals, demonstrating good anti-inflammatory and antioxidant effects in *in vitro* experiments.

Another mechanism is based on the property of certain materials to undergo protonation in an acidic microenvironment. For instance, cationic polymers undergo protonation under acidic conditions, leading to changes in their stability, surface charge, and morphology, thereby promoting drug release from the hydrogel. Yu et al. (2023) prepared a hydrogel microsphere of ZIF-8 nanoparticles coated with itaconic acid (IA) (IA-ZIF-8@HMs), which has both pH responsiveness and protonation-acid responsiveness (Figure 4b). IA can regulate joint inflammation and intracellular oxidative stress levels. The GelMA hydrogel microspheres (HMS) prepared by a one-step microfluidic technique have uniform sizes and can remain suspended in synovial fluid for a long time. *In vitro* experiments show that IA-ZIF-8@HMs can effectively inhibit H_2_O_2_-induced oxidative stress, and IA-ZIF-8 nanoparticles may enter chondrocytes through the complex. After treatment with IA-ZIF-8 nanoparticles and IA-ZIF-8@HMs, the levels of inflammatory factors in the cell supernatant decreased significantly, indicating the potential of this hydrogel for *in vivo* OA treatment. Zhang Z. et al. (2024) developed an injectable hydrogel drug delivery system based on V2C MXenzyme NS, metformin and DexMA for OA treatment (Figure 4c). The MXenzyme hybrid hydrogel could intelligently release metformin in response to pH. Moreover, it scavenged excess reactive oxygen species (ROS) in chondrocytes, alleviated oxidative stress, protected mitochondrial function.

pH-responsive hydrogels can increase the concentration of drugs at the lesion site in OA treatment and reduce side effects on normal tissues. However, the complexity of the joint microenvironment may affect the performance of hydrogels, and their long-term stability and safety still need further evaluation (Dong et al., 2022). On the other hand, current methods for monitoring and regulating pH changes in the joint microenvironment are not precise enough, which may affect the drug-release effect of hydrogels and the consistency of treatment outcomes. Therefore, in the future, it is necessary to conduct further in-depth research on the performance changes of pH-responsive hydrogels in complex *in vivo* environments and develop more precise monitoring and regulation techniques to promote their widespread application in clinical OA treatment.

#### 4.2.3 Enzyme-responsive hydrogels

A prominent feature of OA is the damage and degradation of articular cartilage, which is mainly attributed to the excessive production of various degrading enzymes in the body. Among these enzymes, matrix metalloproteinases (MMPs) are key enzymes in the pathological environment of OA. Their abnormally high expression and activity play a crucial role in the destruction of articular cartilage (Ryplida et al., 2023). Based on these characteristics of MMPs, researchers have developed numerous MMP-responsive drug-hydrogel delivery platforms aiming at the effective treatment of OA. Li et al. (2022) prepared G5-AHP/miR-140 nanoparticles and loaded them into MMP-responsive microspheres (HMS) based on injectable GelMA. The experimental results showed that after injecting this “nano-micro” composite gene hydrogel into the joint cavity of an OA model, HMS could respond to MMPs and achieve the long-term release of G5-AHP/miR-140 nanoparticles. G5-AHP/miR-140 nanoparticles could increase the expression of type II collagen (COL2) in OA chondrocytes.

**FIGURE 5:**
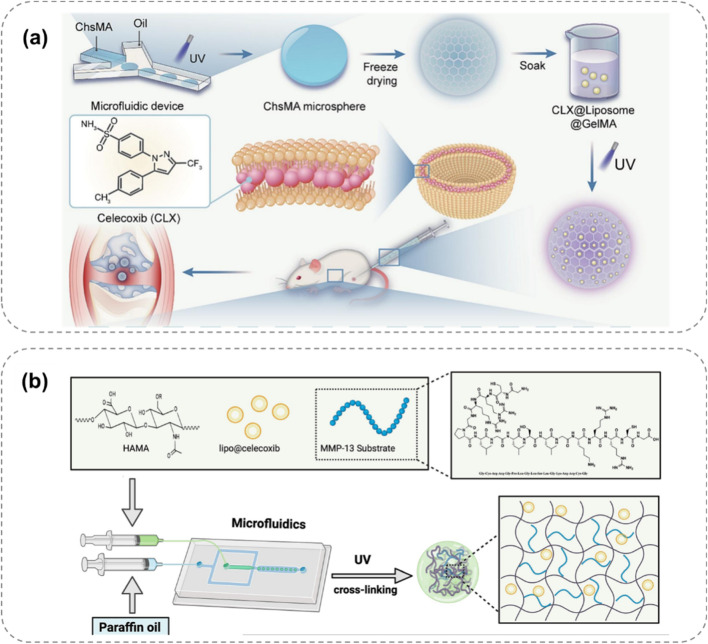
Enzyme-responsive hydrogel systems for targeted OA therapy, showing MMP-triggered drug release and cartilage repair. **(a)** Bilayer ChsMA + CLX@Lipo@GelMA microspheres: MMPs degrade GelMA, releasing CLX (anti-inflammatory) and ChsMA (cartilage repair) (Miao et al., 2024). **(b)** MMP13-responsive HA methacrylate microspheres degrade in high MMP13 environments, releasing celecoxib-loaded liposomes for precise anti-inflammation (Xiang et al., 2024).

Inspired by the dissolution of the chocolate-peanut shell structure, Miao et al. (2024) designed a bilayer microsphere (ChsMA + CLX@Lipo@GelMA) using microfluidic technology, which can respond to the OA microenvironment (Figure 5a). The outer layer of this bilayer microsphere is GelMA, which can rapidly react and degrade in the presence of MMPs in the OA microenvironment, releasing the CLX-loaded liposomes. CLX then exerts an anti-inflammatory effect. The core structure containing ChsMA microspheres is exposed and begins to degrade after the outer shell degrades, releasing chondroitin sulfate to promote the repair of degeneratively damaged OA cartilage. Based on the changes of MMPs in the OA microenvironment, this bilayer microsphere can intelligently release two drugs sequentially, achieving a better therapeutic effect and showing a promising application prospect in disease treatment.

Roy et al. (2024) proposed an enzyme-responsive hydrogel that can continuously release 1H-Indole-2-carboxylic acid, 5-[3-[[[(4-carboxyphenyl) methyl] amino] carbonyl]-1-methyl-1H-pyrazol-5-yl]-, 2-ethyl ester (BI-4394) on demand. BI-4394 can efficiently and specifically block the activity of MMP-13. The researchers prepared a stable and biocompatible hydrogel using glyceryl monostearate TG-18. This hydrogel contains an enzyme-cleavable linker that decomposes under the action of enzymes present in the inflammatory environment, enabling the continuous release of the drug. Xiang et al. (2024) developed an MMP13-responsive micro-nano hydrogel microsphere system for delivering the COX-2 inhibitor celecoxib (Figure 5b). MMP13 is a key enzyme in OA development, and its expression varies during OA progression. The system consists of celecoxib-loaded cationic liposomes non-covalently attached within the microsphere. In an OA environment with high MMP13 expression, the microspheres degrade via the action of MMP13 substrate peptide, facilitating the release of drug-loaded liposomes. Compared to a control solution, the prepared hyaluronic acid methacrylate microspheres showed rapid degradation in an MMP13-containing solution, demonstrating specific enzyme responsiveness for precise anti-inflammatory drug release.

Although enzyme-responsive hydrogels show advantages such as high specificity and biodegradability in OA treatment, there are still some limitations. On one hand, the types, quantities, and activity changes of pathogenic enzymes during the progression of OA are complex, and research in this area is still in its initial stage. This limits the types of pathogenic enzymes that can be designed for enzyme-responsive hydrogels (Lan et al., 2020). On the other hand, the variability of enzyme activity and the time lag in the hydrogel’s response to enzymes may affect the drug-release efficiency and the timeliness of the treatment effect. Therefore, in the future, it is necessary to conduct further in-depth research on the change laws of enzymes in OA and optimize the design of hydrogels to improve their response speed and stability to enzymes, so as to better exert the potential of enzyme-responsive hydrogels in OA treatment (Yu et al., 2021).

#### 4.2.4 Multi-responsive hydrogels

**FIGURE 6:**
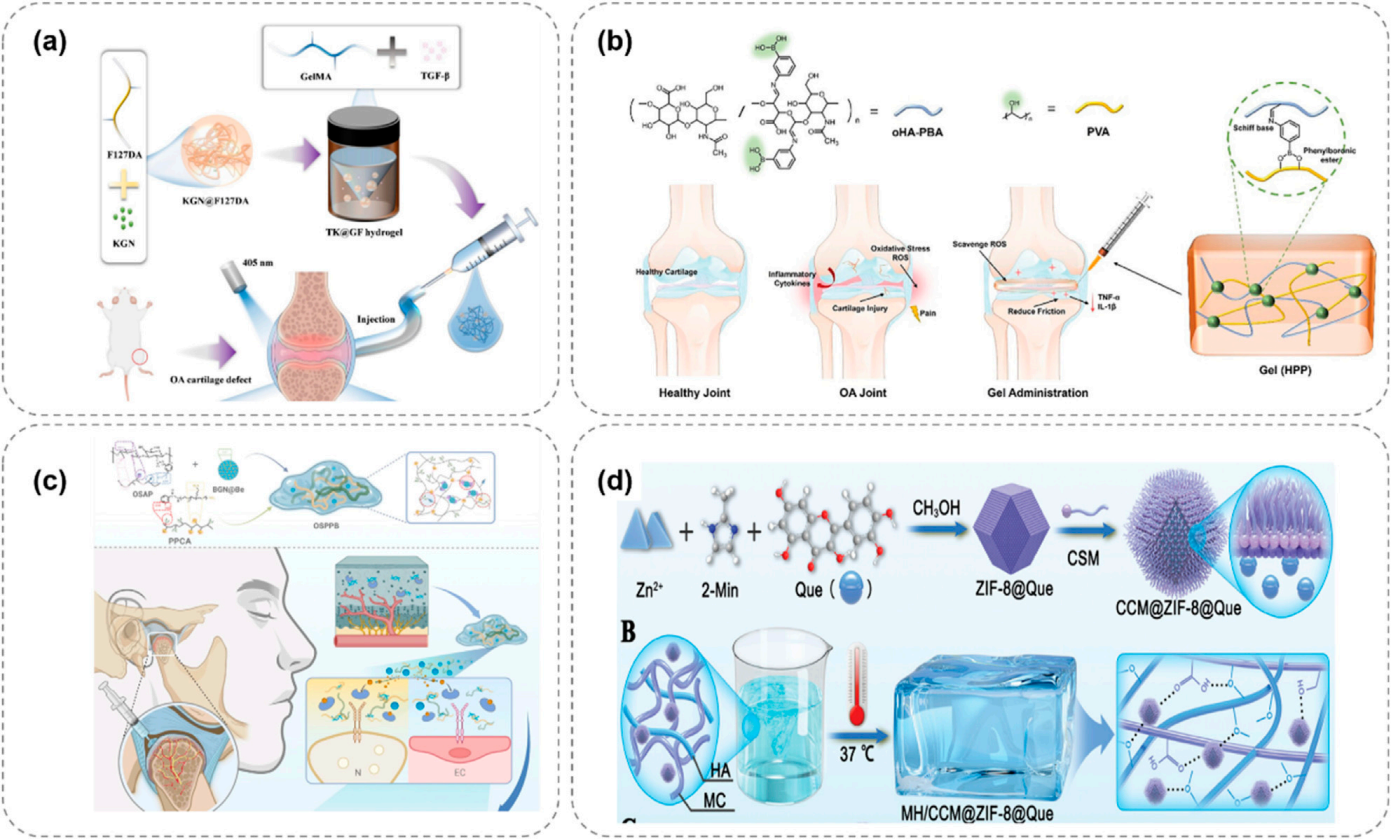
Multi-responsive hydrogel systems for OA, integrating temperature, pH, and ROS responsiveness to enhance therapeutic precision. **(a)** Thermo/enzyme-responsive hydrogel releases drugs via temperature rise and enzyme activation, boosting OA treatment efficacy (Sun et al., 2025). **(b)** pH/ROS-responsive APBA-SF/PVA hydrogel releases IFN-γ under OA’s acidity and high ROS, reducing inflammation (Lei et al., 2024). **(c)** Dual-responsive OSPPB hydrogel inhibits neurovascularization in OA via stimulus-triggered drug release (Qin et al., 2025). **(d)** ZIF-8-based hydrogel responds to pH/ROS, releasing quercetin to remodel OA’s microenvironment and promote repair (Salama et al., 2025).

Hydrogels with a single-response mechanism have certain limitations in the treatment of OA. To more precisely mimic the complex pathological microenvironment of OA and achieve more efficient treatment, multi-responsive hydrogels have emerged. These hydrogels integrate multiple response mechanisms and can simultaneously respond to multiple stimuli, enabling more intelligent and effective drug delivery and therapeutic effects (Jahanbekam et al., 2023). Some studies have combined temperature-responsive and enzyme-responsive mechanisms to prepare hydrogels with dual-response characteristics. For example, in certain hydrogel systems, a phase transition occurs in response to temperature changes, enabling preliminary regulation of drug release. Meanwhile, when exposed to excessive enzymes in the OA microenvironment, the drug release can be further accelerated (Figure 6a; Sun et al., 2025). This design ensures that at the site of joint inflammation, the hydrogel can not only release drugs due to a rise in temperature but also replenish the drugs in a timely manner when the enzyme concentration increases, enhancing the therapeutic effect.

There are also studies on hydrogels that are responsive to both pH and reactive oxygen species (ROS). During the pathological process of OA, the joint microenvironment is not only acidic but also has a significantly elevated ROS level. Lei et al. (2024) designed a hydrogel composed of 3-aminophenylboronic acid-modified silk fibroin (APBA-SF) and polyvinyl alcohol (PVA), which was loaded with interferon-γ (IFN-γ) microbubbles (Figure 6b). The boronate ester bonds in this hydrogel are responsive to ROS and can break when the ROS concentration increases, releasing IFN-γ. At the same time, in the acidic OA microenvironment, the structure of the hydrogel also changes, further promoting drug release. Qin et al. (2025) developed a hydrogel (OSPPB) formed by cross-linking aldehyde-phenylboronic acid-modified sodium alginate/polyethyleneimine-grafted protocatechuic acid and bevacizumab-sustained-release nanoparticles (Figure 6c). Zhang et al. (Salama et al., 2025) designed a lubricating and dual-responsive injectable hydrogel (MH/CCM@ZIF-8@Que) based on ZIF – 8 impregnated with Quercetin (Figure 6d). The hydrogel exhibits pH and reactive oxygen species (ROS) responsiveness and can controllably release bioactive substances to modulate the bone tissue microenvironment. It mitigates synovitis, cartilage matrix degeneration, and promotes cartilage repair.

The advantage of multi-responsive hydrogels is that they can more comprehensively respond to various changes in the OA microenvironment, improving the precision of drug delivery and the durability of the therapeutic effect (Bhattacharjee et al., 2022). However, their design and preparation also face many challenges. The integration of multiple response mechanisms may make the structure and properties of the hydrogel complex and difficult to precisely control. There may be interference between different response mechanisms, affecting the accuracy and stability of the hydrogel’s response to stimuli (Yu et al., 2022). In addition, there is relatively little research on the long-term stability and biosafety of multi-responsive hydrogels in the complex *in vivo* environment, which limits their application in clinical treatment.

### 4.3 Mechanical and viscoelastic properties

Injectable hydrogels for OA treatment must meet specific mechanical and viscoelastic criteria to adapt to the dynamic joint environment. Load-bearing capacity is critical: hydrogels should mimic the compressive modulus of native articular cartilage (0.5–10 MPa) to withstand cyclic joint loading, preventing premature deformation or failure. For example, composite hydrogels reinforced with nanocellulose exhibit enhanced compressive strength, maintaining structural integrity under repetitive motion (Chen et al., 2022).

Self-healing ability enables hydrogels to repair microdamage caused by joint movement. This property, often mediated by dynamic bonds (e.g., metal coordination or hydrogen bonds), allows hydrogels to reassemble their network after shear or compressive stress—an essential feature for long-term efficacy (Goncu and Ay, 2023). A recent study showed that a chitosan-hyaluronic acid hydrogel with self-healing properties retained 80% of its mechanical strength after 1,000 cycles of compression, outperforming non-healing counterparts in OA models (Wu D. et al., 2024). Lubricating properties are equally vital, as reduced friction alleviates joint stiffness and pain. Hydrogels incorporating hyaluronic acid or phospholipids mimic the lubricity of synovial fluid (coefficient of friction <0.01), improving mobility by minimizing cartilage-on-cartilage wear (Guo et al., 2025). For instance, HA-based hydrogels with surface-grafted lubricin exhibit superior lubrication, reducing friction-induced cartilage damage *in vitro* (von Lospichl et al., 2017). These mechanical and viscoelastic attributes—load-bearing capacity, self-healing, and lubrication—are thus integral to the design of effective intra-articular hydrogels, ensuring they not only deliver therapeutics but also directly mitigate OA-related mechanical dysfunction.

### 4.4 Injectable hydrogels as cell delivery platforms for cartilage regeneration

A critical application of injectable hydrogels in OA treatment lies in their role as cell delivery platforms for regenerative therapies, supporting stem cell or chondrocyte transplantation to restore damaged cartilage (Lei and Fan, 2025). These hydrogels act as biomimetic scaffolds that mimic the native extracellular matrix (ECM), providing a favorable microenvironment for cell viability, proliferation, and chondrogenic differentiation.

Cell viability maintenance is foundational: hydrogels with optimal porosity and interconnected networks facilitate nutrient diffusion and waste removal, ensuring >80% cell survival over 4 weeks *in vitro* (Liu et al., 2021). For example, a hyaluronic acid-collagen hydrogel loaded with mesenchymal stem cells (MSCs) maintained 90% viability in a rabbit OA model, outperforming cell suspensions alone (Zhu J. et al., 2022). Chondrogenic differentiation is guided by hydrogel composition and bioactive cues. Incorporating chondrogenic growth factors (e.g., TGF-β3) or ECM-derived peptides (e.g., RGD) into hydrogels promotes MSC differentiation into chondrocytes, as evidenced by increased expression of type II collagen and aggrecan (Sang et al., 2022). A recent study showed that a silk fibroin hydrogel functionalized with kartogenin enhanced chondrogenesis, with differentiated cells exhibiting a phenotype similar to native chondrocytes (Wang P. et al., 2024). Integration with host tissue is key for long-term repair. Hydrogels modified with adhesion peptides (e.g., GFOGER) improve cell-matrix and matrix-tissue interactions, enabling seamless integration with surrounding cartilage. In a murine OA model, an injectable hydrogel delivering chondrocytes showed 30% higher integration efficiency compared to non-modified hydrogels, reducing the risk of graft detachment (Zhao et al., 2024).

## 5 Challenges and perspectives

Despite significant progress in the field of injectable hydrogels for OA treatment, numerous challenges remain before their widespread clinical application can be achieved. In terms of material properties, many current hydrogels lack self-healing capabilities. During the injection process or due to joint movement, they may suffer mechanical damage under external forces, affecting their long-term stability within the joint and treatment efficacy (Ying et al., 2023). Regarding clinical translation, most existing studies are still at the pre-clinical rodent model stage, lacking sufficient human clinical trial data to verify their efficacy and safety in OA patients (Wang X. et al., 2024). Due to the differences in the physiological structure of human joints and the pathological microenvironment compared with animal models, it is difficult to directly extrapolate the results of animal experiments to humans. For example, hydrogels that perform well in animal experiments may encounter issues such as immune responses and inconsistent drug-release rates in human clinical trials.

Furthermore, the specific mechanism of action of hydrogels in treating OA has not been fully elucidated, and the optimal treatment time window remains to be further investigated and determined (Tnibar, 2022). This makes it difficult to precisely develop treatment plans in clinical applications, preventing the full exploitation of the therapeutic potential of hydrogels. Meanwhile, there is still much room for optimizing the injection conditions of current hydrogels. Ensuring that hydrogels can accurately reach the diseased site within the joint and distribute evenly is an important issue that needs to be addressed (Chen J. et al., 2025). Additionally, the compatibility between the properties of hydrogels and the implantation site needs to be further improved to adapt to the individual differences and lesion characteristics of different patients.

Looking to the future, to overcome these challenges, in the aspect of clinical translation, more high-quality clinical trials are needed to accumulate more data for evaluating the efficacy and safety of hydrogels. At the same time, advanced imaging techniques and biological monitoring methods can be utilized to track the *in vivo* behavior of hydrogels in real-time, including drug release, degradation processes, and interactions with surrounding tissues, providing a basis for optimizing treatment plans. In optimizing injection conditions and the compatibility between materials and implantation sites, advanced technologies such as 3D printing can be combined. 3D printing offers unique advantages in the field of injectable hydrogels by enabling personalized design and precise fabrication of hydrogel structures tailored to individual joint anatomy. For example, based on patient-specific imaging data (e.g., MRI or CT scans), 3D printing can pre-fabricate hydrogel scaffolds with customized porosities, mechanical properties, and drug-loading patterns that match the size and shape of the OA-affected joint area (Shi et al., 2022). These pre-printed structures can then be formulated into injectable formulations (e.g., shear-thinning hydrogels or particle suspensions) that retain their designed architecture post-injection, ensuring targeted drug delivery and enhanced integration with host tissues. Additionally, 3D printing allows for the layer-by-layer incorporation of multiple components (e.g., growth factors, cells, or responsive nanoparticles) within the hydrogel matrix, enabling spatiotemporally controlled release of therapeutics to address the multifaceted pathology of OA (Behan et al., 2022). This level of customization and precision is difficult to achieve with traditional bulk hydrogel preparation methods, making 3D printing a promising tool to improve the efficacy and specificity of injectable hydrogel-based therapies (Zha et al., 2023). For example, using the imaging data of patients, 3D printing technology can be used to prepare hydrogel carriers that are highly matched to the shape of the patient’s joint and the diseased site, improving the precision of drug delivery.

## 6 Conclusion

In conclusion, we have comprehensively explored the application of injectable hydrogels in the treatment of OA. Hydrogels, whether natural polymer-based, synthetic polymer-based, or composite, offer unique advantages such as biocompatibility, biodegradability, and the ability to control drug release. Different cross-linking strategies, including chemical, physical, and multiple cross-linking, endow hydrogels with diverse properties, enabling them to meet various treatment requirements. Thermosensitive, pH-responsive, enzyme-responsive, and multi-responsive hydrogels have been developed to target the specific pathological features of OA, such as elevated joint temperature, acidic microenvironment, and increased enzyme levels. These responsive hydrogels can achieve intelligent drug delivery, effectively inhibit cartilage degradation, and reduce inflammation, showing great potential in OA treatment. However, several challenges need to be addressed before the widespread clinical use of injectable hydrogels for OA. The lack of self-healing capabilities in many hydrogels may affect their long-term stability in joints. Insufficient human clinical trial data and the unclear mechanism of action limit the precise development of treatment plans. Looking ahead, more high-quality clinical trials are essential to evaluate the efficacy and safety of hydrogels. Advanced imaging and monitoring techniques can help track the *in vivo* behavior of hydrogels. Moreover, combining technologies like 3D printing can optimize injection conditions and improve the compatibility between hydrogels and implantation sites. Overall, injectable hydrogels hold great promise for the treatment of OA, and with continuous research and innovation, they may become an effective treatment option in the future.
